# Comparative metabolite profiling of salt sensitive *Oryza sativa* and the halophytic wild rice *Oryza coarctata* under salt stress

**DOI:** 10.1002/pei3.10155

**Published:** 2024-06-15

**Authors:** Nishat Tamanna, Anik Mojumder, Tomalika Azim, Md Ishmam Iqbal, Md Nafis Ul Alam, Abidur Rahman, Zeba I. Seraj

**Affiliations:** ^1^ Plant Biotechnology Laboratory, Department of Biochemistry and Molecular Biology University of Dhaka Dhaka Bangladesh; ^2^ Center for Bioinformatics Learning Advancement and Systematic Training University of Dhaka Dhaka Bangladesh; ^3^ Department of Genetic Engineering and Biotechnology University of Dhaka Dhaka Bangladesh; ^4^ Department of Biochemistry and Microbiology North South University Dhaka Bangladesh; ^5^ Arizona Genomics Institute, School of Plant Sciences The University of Arizona Tucson Arizona USA; ^6^ Department of Plant Biosciences, Faculty of Agriculture Iwate University Morioka Japan; ^7^ Department of Plant Sciences, College of Agriculture and Bioresources University of Saskatchewan Saskatoon Saskatchewan Canada

**Keywords:** halophyte, metabolomics, *Oryza coarctata*, *Oryza sativa*, Rice, salinity

## Abstract

To better understand the salt tolerance of the wild rice, *Oryza coarctata*, root tissue‐specific untargeted comparative metabolomic profiling was performed against the salt‐sensitive *Oryza sativa*. Under control, *O. coarctata* exhibited abundant levels of most metabolites, while salt caused their downregulation in contrast to metabolites in *O. sativa*. Under control conditions, itaconate, vanillic acid, threonic acid, eicosanoids, and a group of xanthin compounds were comparatively abundant in *O. coarctata*. Similarly, eight amino acids showed constitutive abundance in *O. coarctata*. In contrast, under control, glycerolipid abundances were lower in *O. coarctata* and salt stress further reduced their abundance. Most phospholipids also showed a distribution similar to the glycerolipids. Fatty acyls were however significantly induced in *O. coarctata* but organic acids were prominently induced in *O. sativa*. Changes in metabolite levels suggest that there was upregulation of the arachidonic acid metabolism in *O. coarctata*. In addition, the phenylpropanoid biosynthesis as well as cutin, suberin, and wax biosynthesis were also more enriched in *O. coarctata*, likely contributing to its anatomical traits responsible for salt tolerance. The comparative variation in the number of metabolites like gelsemine, allantoin, benzyl alcohol, specific phospholipids, and glycerolipids may play a role in maintaining the superior growth of *O. coarctata* in salt. Collectively, our results offer a comprehensive analysis of the metabolite profile in the roots of salt‐tolerant *O. coarctata* and salt‐sensitive *O. sativa*, which confirm potential targets for metabolic engineering to improve salt tolerance and resilience in commercial rice genotypes.

## Graphical abstract

To gain a better insight of the remarkable salt tolerance of the wild rice, *O. coarctata*, root tissue specific untargeted comparative metabolomic profiling was performed against the salt sensitive *O. sativa*. Strikingly, under control condition, *O. coarctata* displayed an abundance in most metabolites including itaconate, vanillic acid, threonic acid, eicosanoids, a group of xanthin compounds and phospholipids, while salt caused a shift towards their downregulation in contrast to the fate of metabolites in *O. sativa*. Our results provide a comprehensive analysis of the metabolite pool in the roots of salt tolerant *O. coarctata* and salt sensitive *O. sativa*, and identify some of the potential pathways that can be targeted for future metabolic engineering to improve salt tolerance and resilience in commercial rice genotypes.

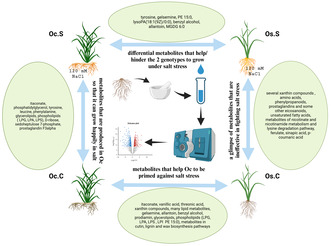

## 1 INTRODUCTION

Crop yield is highly susceptible to various abiotic stresses, including salinity. Salt stress is currently one of the major limiting factors for crop production and poses a growing threat (Afzal et al., 2022). It is estimated that 20% of cultivated land and 33% of irrigated land have high salinity worldwide, with further exacerbation due to the effect of climate change (Shrivastava & Kumar, 2015). Rice (*Oryza sativa* L.) is a salt‐sensitive staple food for more than half of the world's population, particularly in Asia, the Middle East, Latin America, and the West Indies (Fukagawa & Ziska, 2019). Given the increase in the proportion of salt‐afflicted cropland, it will be harder to maintain the production of rice to feed the growing population. Hence, unraveling the mechanisms by which specific plants defend against salt stress while continuing to grow satisfactorily will help to design better rice for growth in saline‐affected farmlands.

*O. sativa* is a glycophyte and highly sensitive to salt stress (Hoang et al., 2016). Rice is unable to withstand a threshold of 3 dS/m salt, while soil is only considered saline if electrical conductivity (EC) exceeds 4 dS/m (Singh et al., 2021). Plants are subject to two phases of stress due to salinity: osmotic and ionic. The initial osmotic phase under saline conditions later progresses into the ionic stress phase after salt accumulation, resulting in leaf cell death. Rice landraces evolving in coastal areas prone to salt stress employ various stress response mechanisms to ensure survival. In high‐yielding modern genotypes, any defense against salt stress is achieved at the expense of a significant decrease in yield. For example, it has been reported that even at an EC as low as 3.5 dS/m, there is a 10% yield loss, and this further increases to 50% at an EC of 7.2 dS/m (Hoang et al., 2016). The drastic effects of salinity on the growth and physiology of *O. sativa* are especially pronounced during its seedling and reproductive stages (Singh et al., 2021). On the other hand, the halophytic wild rice species *Oryza coarctata* has a remarkable ability to resist salt stress and can complete its life cycle under relatively high salt concentrations (>400 mM or >40 dS/m), and even set a few grains per panicle (Ishikawa et al., 2022). Popularly referred to as Asian wild rice, its natural habitats are the highly saline coastal regions of Southeast Asian countries (Mondal et al., 2018).

*O. coarctata* is an allotetraploid with a unique KKLL genome (Lu et al., 2009). The morphology of *O. coarctata* differs significantly from *O. sativa*. It has a highly differentiated rhizomatous system from which leafy shoots and rhizoid‐like rootlets emerge (Figure S1). The rootlets account for a sturdier foothold and improved absorption, especially during saline stress (Sengupta & Majumder, 2010). *O. coarctata* is consistently able to maintain a low Na^+^: K^+^ ratio in the leaf, even under increasing salt stress conditions. An important factor in its salt establishment is the ability of micro hairs in the waxy leaves of *O. coarctata* to secrete salt (Sengupta & Majumder, 2010; Somasundaram et al., 2020). These specialized salt glands on the leaf adaxial and abaxial surfaces contribute to a low Na^+^:K^+^ ratio, with their enhanced performance in proportion to the increase in levels of salinity (Sengupta & Majumder, 2010). The presence of Na^+^ transporters proximal to the xylem vessels also helps alleviate Na^+^ accumulation in their shoots (Somasundaram et al., 2020). Other findings indicate that *O. coarctata* also prevents passive entry of Na^+^ into root cells, while *O. sativa* depends heavily on the Na^+^/H^+^ SOS1 exchanger activity to excrete out Na^+^ (Ishikawa et al., 2022).

The levels of metabolites in plants are influenced by both biological and environmental factors, and hence metabolomic approaches have great potential to bridge the knowledge gap between genotype and phenotype. Untargeted metabolomics, also called global metabolomics, is based on the discovery and quantitation of metabolites at the organism scale (Schrimpe‐Rutledge et al., 2016). Several research groups have used metabolomic profiling in different species to compare contrasting genotypes and to elucidate the underlying mechanisms that cause their adaptation or susceptibility to very high levels of salt stress (Al Kharusi et al., 2021; Niron et al., 2020).

In rice, metabolomic approaches have also been widely used to study salt tolerance mechanisms. Gupta and De (2017) analyzed the differences in metabolite profiles between 4 varieties of indica rice: 2 salt‐sensitive (Sujala and MTU 7029) and 2 tolerant (Bhutnath and Nonabokra) under salt stress conditions (Gupta & De, 2017). Their findings showed increased production of the signaling molecules, serotonin, and gentisic acid in leaves and they proposed that these compounds may be contributing to NaCl tolerance. In comparison with the sensitive rice genotype, IR64, the tolerant FL478 was shown to accumulate more metabolites to combat the osmotic stress at the latter phases of salt stress. In contrast, a rapid decrease in organic acids was observed in the tolerant genotype at the onset of stress. (Zhao et al., 2014). Genomic sequencing, transcriptomic sequencing, and proteomic analysis have been performed on *O. coarctata* to understand its underlying salt tolerance mechanisms (Garg et al., 2014; Mondal et al., 2018; Sengupta & Majumder, 2010; Zhao et al., 2023). Differential gene expression analyses from deep transcriptomic study of *O. coarctata* under control versus low‐ and high‐saline conditions revealed key metabolic pathways involved in its stress tolerance (Garg et al., 2014). This study found the stress‐induced transcription of *O. coarctata* to be enhanced, including several transcription factors, key enzymes of important metabolic pathways, secondary metabolites such as hydroxycinnamic acid, serotonin amides, and phenylpropanoids, the ethylene biosynthesis genes, suberin and cellulose, etc. For the proteomics study, two‐dimensional gel electrophoresis and MALDI–TOF techniques were used to compare *O. coarctata* and *O. sativa* under salinity stress which showed differences in the proportion of key proteins. Notably, CP47 protein, CRT/DRE‐binding protein, Chloroplastic Heat‐shock protein 70, Cellulose synthase‐like protein, and Alcohol dehydrogenase 1 exhibited remarkable upregulation in *O. coarctata* at 400 mM salinity. In addition, the study emphasized the potential roles of Manganese stabilizing protein (MSP), PS1 reaction center subunit IV, RuBisCO, and RuBisCO activase, the chloroplastic precursor of glutamine synthase, L‐myo‐inositol 1‐phosphate synthase, and sucrose synthase in conferring salinity tolerance (Sengupta & Majumder, 2009). Other studies compared the different strategies used by *O. coarctata* and *O. sativa* to control cellular Na^+^ and K^+^ homeostasis (Ishikawa et al., 2022; Ishikawa & Shabala, 2019).

The susceptibility or tolerance of plants to high‐saline conditions is dependent on the timely coordination of multiple genes and pathways (Tuteja, 2007). As there are multiple factors in play, integration of multi‐omics data is necessary to obtain a complete picture. Untargeted metabolomics allows a good understanding of changes in metabolism due to stress at the tissue level in real time. In addition, most of the rice metabolomic studies have focused on leaves, while plants encounter salt stress first in the root (Lawas et al., 2019; Yan et al., 2022). We performed comparative untargeted/global metabolomics using root tissues of salt‐sensitive *O. sativa* and highly salt‐tolerant *O. coarctata* under control (0 mM NaCl) and saline (120 mM NaCl) conditions to reduce the gap in the knowledge of root‐specific adjustments in metabolites which help or hinder the plant to survive as it encounters abnormal salt levels. Our findings corroborate that *O. coarctata* possesses a comprehensive metabolic defense strategy to thrive under salt stress. It exhibited higher levels of key metabolites involved in osmotic adjustment, antioxidant defense, and energy generation. In addition, *O. coarctata* showed consistent stability in nicotinate and nicotinamide metabolism, while *O. sativa* displayed distinct alterations under salt stress. These findings confirm specific metabolites that could play pivotal roles in the response to salt stress in the roots of *O. coarctata*, and offer valuable insights for the development of salttolerant crops.

## 2 RESULTS

### 2.1 Metabolic profiles of O. sativa and O. coarctata under salt stress

*Oryza coarctata*, a halophytic distant relative of the salt‐sensitive glycophyte *O. sativa*, exhibits an extremely high salt tolerance capacity. The comparative analytic study of the metabolites of the roots of *O. coarctata* and *O. sativa* with and without salt by LC–MS identified a total of 1012 metabolites (File S1). The accumulated metabolites in the roots of the two species included different fatty acids, amino acids, organic acids, sugars, amines, and glycerophospholipids.

**FIGURE 1:**
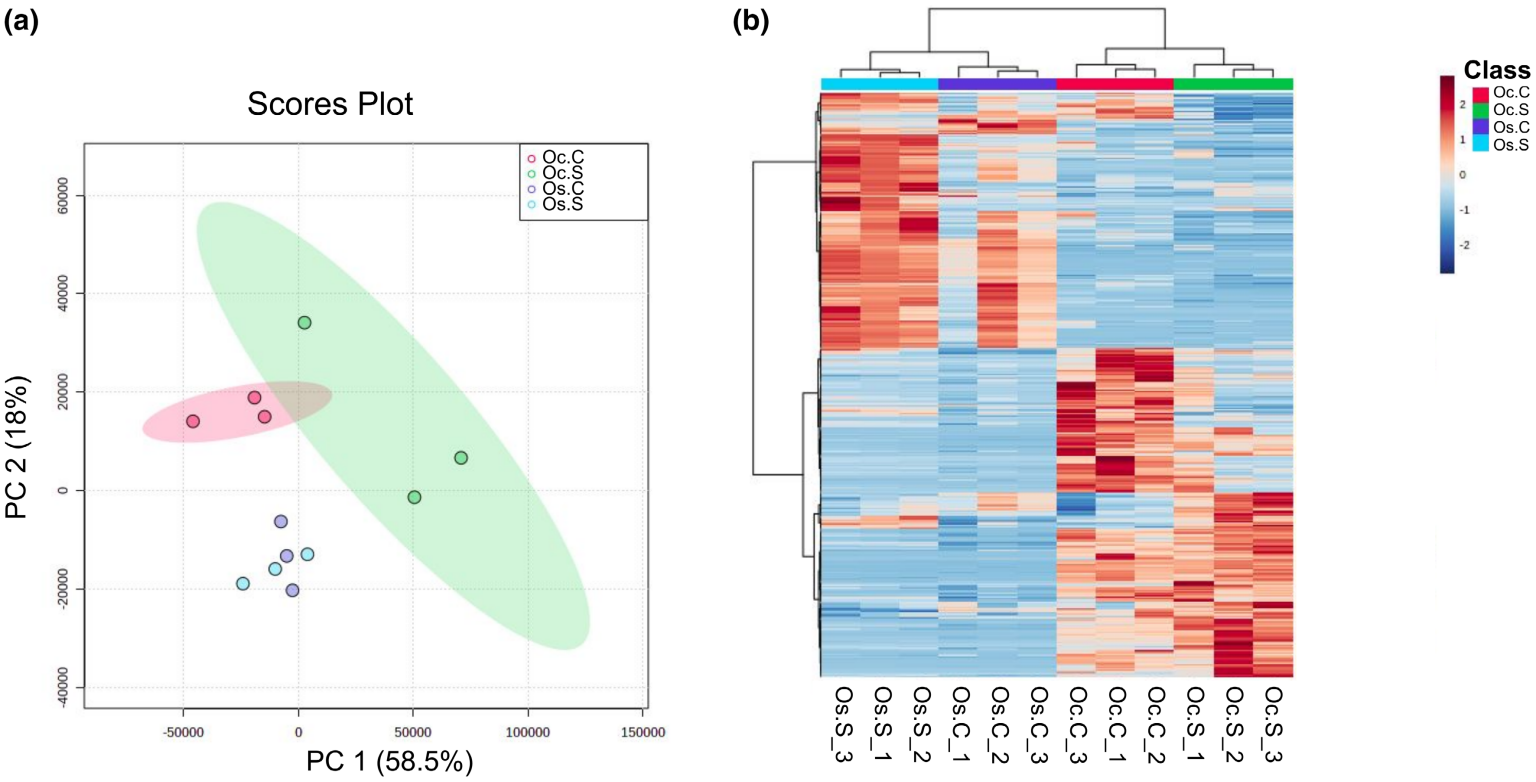
(a) Score plot from PCA analysis of metabolite profiles of *Oryza sativa* and *Oryza coarctata* without and with salt stress samples. Red (left) and green (right) ellipsoids show a 95% confidence interval in *Oryza coarctata* without stress and with stress plants, respectively. (b) Hierarchical clustering for the top 500 metabolites of *Oryza sativa* and *Oryza coarctata* without and with salt stress samples. The columns represent the samples, whereas the rows represent the metabolites. Higher and lower concentrations of metabolites are indicated by an increase in the intensities of red and blue, respectively. Three biological replicates were performed.

Principal component analysis was performed to get a global view of the metabolite changes in these two contrasting rice species with or without salt. There is a clear separation between the metabolite clusters of the control and salt‐stressed samples in both species (Figure 1a). Hierarchical clustering of the top 500 metabolites based on the highest variations reinforced this observation (Figure 1b). These plots validate the notion that our replicated analyses reliably captured the degree of variation across the conditions for both species.

### 2.2 Salt induces changes in the global metabolite profiling

*O. sativa* (Os) genotypes struggle to survive under salt stress, in particular, the genotype used for this experiment. On the other hand, wild halophytic rice (Oc) thrives under salt stress. Moreover, the latter is the only halophyte that can set rice‐like grains, underscoring its commonality to cultivated rice. For the 1st group of comparison, Oc.C/Os.C under control conditions, we set out to discover whether there are any pre‐existing metabolites in *O. coarctata* (and absent in *O. sativa*) which help the former to prime against any anticipated salt stress, since *O. coarctata* has evolved in salty coastal areas. For the 2nd group of comparison, for wild rice, Oc.S/Oc.C, under control versus salty conditions, we set out to find metabolic pathways that are activated to help *O. coarctata* thrive under salt stress. Similarly in the 3rd comparison between cultivated rice, Os.S/Os.C, which are the metabolites that fail to help *O. sativa* survive well when grown under normal versus salty conditions. For the 4th comparative study on Oc.S/Os.S under salty conditions, the aim was to discover the differential metabolites produced that allow *O. coarctata* to thrive while *O. sativa* struggles to survive. Moreover, we looked at the commonalities and differences in all 4 groups.

**FIGURE 2:**
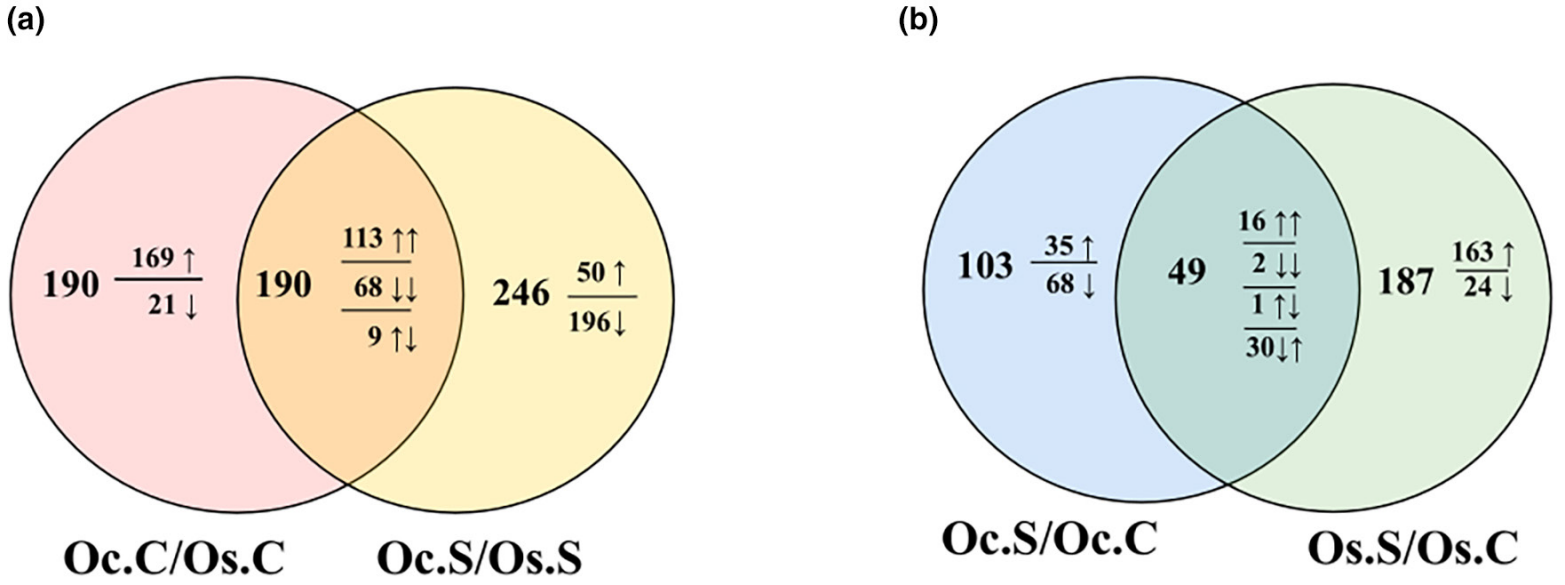
Venn diagrams of differentially accumulated metabolites (|fold change|>1.5 and *p* < .05) revealing commonly or uniquely up‐ (↑) and downregulated (↓) metabolites under control and salt conditions between (a) Oc.C/Os.C and Oc.S/Os.S (b) Oc.S/Oc.C, and Os.S/Os.C comparison groups. The order of arrows in intersecting regions follows the order of the circles.

The investigation of *O. coarctata* and *O. sativa* under control conditions revealed 380 differentially accumulated metabolites (Figure 2a). Upon exposure to 120 mM NaCl for 72 h, this number increased to 436, with only 190 metabolites in common between the two conditions. *O. coarctata* displayed an abundance of most metabolites under control conditions, while salt stress caused their downregulation (File S2).

The response to 120 mM NaCl treatment for 72 hours resulted in significant alterations in metabolite profiles for both genotypes compared to their respective controls (Figure 2b). Among the responding metabolites to NaCl stress, 49 were common for both genotypes, with 103 and 187 metabolites being specific to *O. coarctata* and *O. sativa*, respectively. Interestingly, *O. coarctata* exhibited downregulation of most (100) of its metabolites in response to salt stress, whereas *O. sativa* showed an opposite trend with upregulation of the majority (209) of its metabolites (Figure 2a,b). This contrasting response of metabolites under salt stress in these two rice genotypes suggests that the acquired salt resistance of *O. coarctata* may be a function of a constitutive pool of metabolites as well as linked to the altered metabolite flux. File S3 presents the detailed comparative data from the Venn diagrams in Figure 2a,b.

### 2.3 Differentially accumulated metabolites in the four comparison groups

**FIGURE 3:**
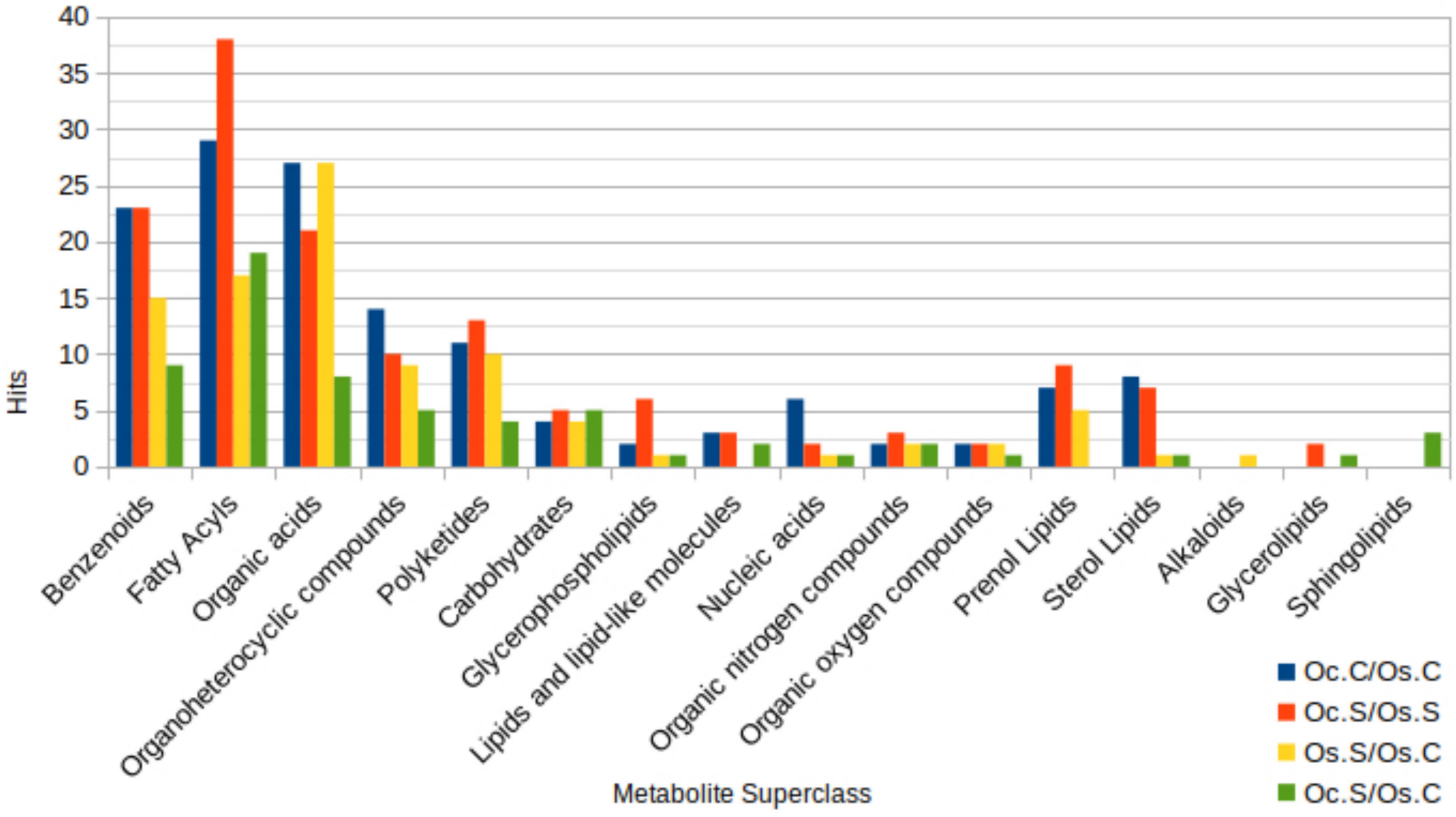
Grouped bar plot indicating enriched metabolite sets analysis in four comparison groups: Oc.C/Os.C, Oc.S/Oc.C, Os.S/Os.C, and Oc.S/Os.S. The number of differentially accumulated metabolites (|fold change|>1.5 and *p* < .05) that enrich a particular super‐class of metabolite sets based on chemical structure for each group accounts for the height of each bar.

Differentially accumulated metabolites in the four comparison groups were further classified into their superclasses (Figure 3). Differential accumulation of fatty acyls, organic acids as well as benzenoids were found in all four groups. The highest number of fatty acyls were differentially accumulated in the Oc.S vs Os.S group, whereas the highest number of organic acid hits were found in the groups – Oc.C vs Os.C and Os.S vs Os.C (Figure 3).

**FIGURE 4:**
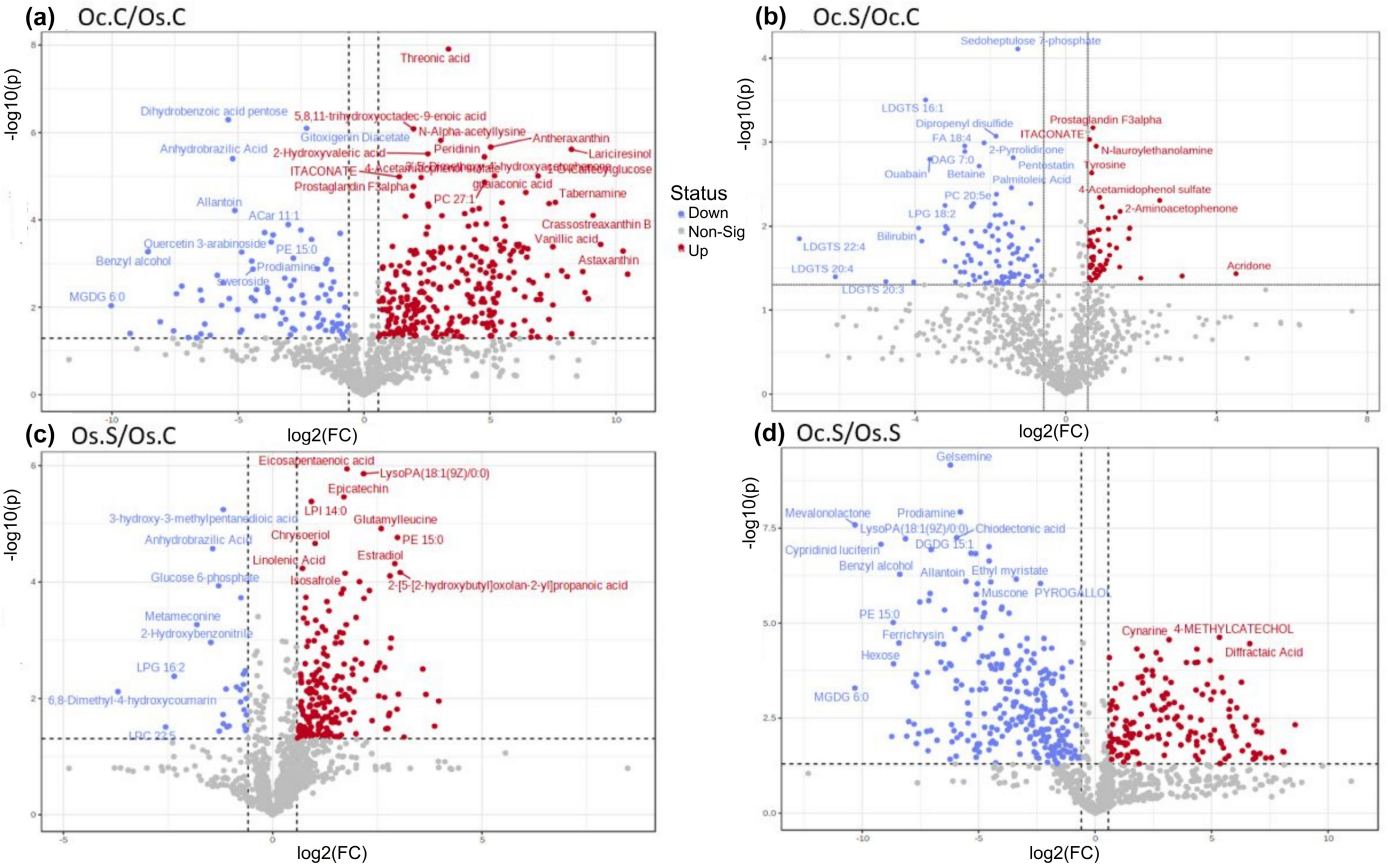
Volcano plots representing significantly modulated metabolites in comparison groups a) Oc.C/Os.C b) Oc.S/Oc.C c) Os.S/Os.C and d) Oc.S/Os.S [analysis cut‐off: |fold change|>1.5 and *p* < 0.05] [red = upregulated; blue = downregulated; grey = nonsignificant].

When the metabolome of *O. coarctata* and *O. sativa* were compared, a significant number of metabolites showed altered expression profiling, as shown in the volcano plot (Figure 4). Here, the red and blue dots depicting up and downregulation, respectively, represent differentially expressed metabolites with FC >1.5 (Fold‐Change) and *p* < .05.

In the case of the 1st group (Oc.C vs. Os.C), itaconate, vanillic acid, threonic acid, a group of xanthin compounds, and many lipid metabolites were found to exist at a higher level in *O. coarctata* compared to *O. sativa* under control conditions (Figure 4a). Among the xanthin compounds, antheraxanthin, astaxanthin, amarouciaxanthin A, cassostreaxanthin B, and nostoxanthin were found in extremely high concentration (32–141⨯) (File S3) in *O. coarctata* compared to *O. sativa*. Comparative analysis of the lipid group between *O. coarctata* and *O. sativa* samples showed a mixed profile of differential accumulation of the fatty acyls. In the glycerolipid group, most of the lysodiacylglyceryltrimethylhomoeserine (LDGTS) had almost no difference, while monogalactosylmonoacylglycerol (MGMG), digalactosylmonoacylglycerol (DGMG), and digalactosyldiacylglycerol (DGDG) were lower in *O. coarctata* compared to *O. sativa* (Figure 6). In the phospholipid group, the majority of the lyso‐phosphatidylglycerol (LPG), lyso‐phosphatidate (LPA) and lyso‐phosphatidylserine (LPS) were in lower concentration in *O. coarctata*. Under control conditions, some other phospholipid groups, like lyso‐phophatidylinositol (LPI) and lyso‐phophatidylcholine (LPC) were mostly found in higher concentration in *O. coarctata* compared to their levels in *O. sativa*. Different Lyso‐phophatidylethanolamines (LPEs) at control were observed to be both up‐ and downregulated in *O. coarctata* (Figure 6).

**FIGURE 5:**
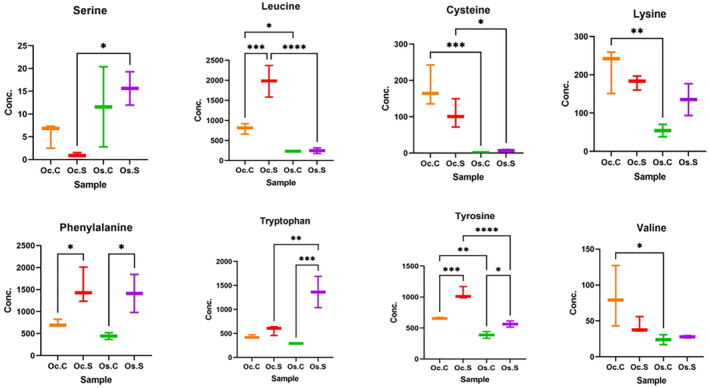
Comparison of amino acid levels in four sample groups: Oc.C, Oc.S, Os.C, and Os.S. Statistical analysis was performed using one‐way ANOVA followed by Šídák multiple comparisons test. *p*<.05 were considered significant. * denotes .01 < *p* < .05, ** denotes .001 < *p* < .01, *** denotes .0001 < *p* < .001, **** denotes *p* < .0001.

**FIGURE 6:**
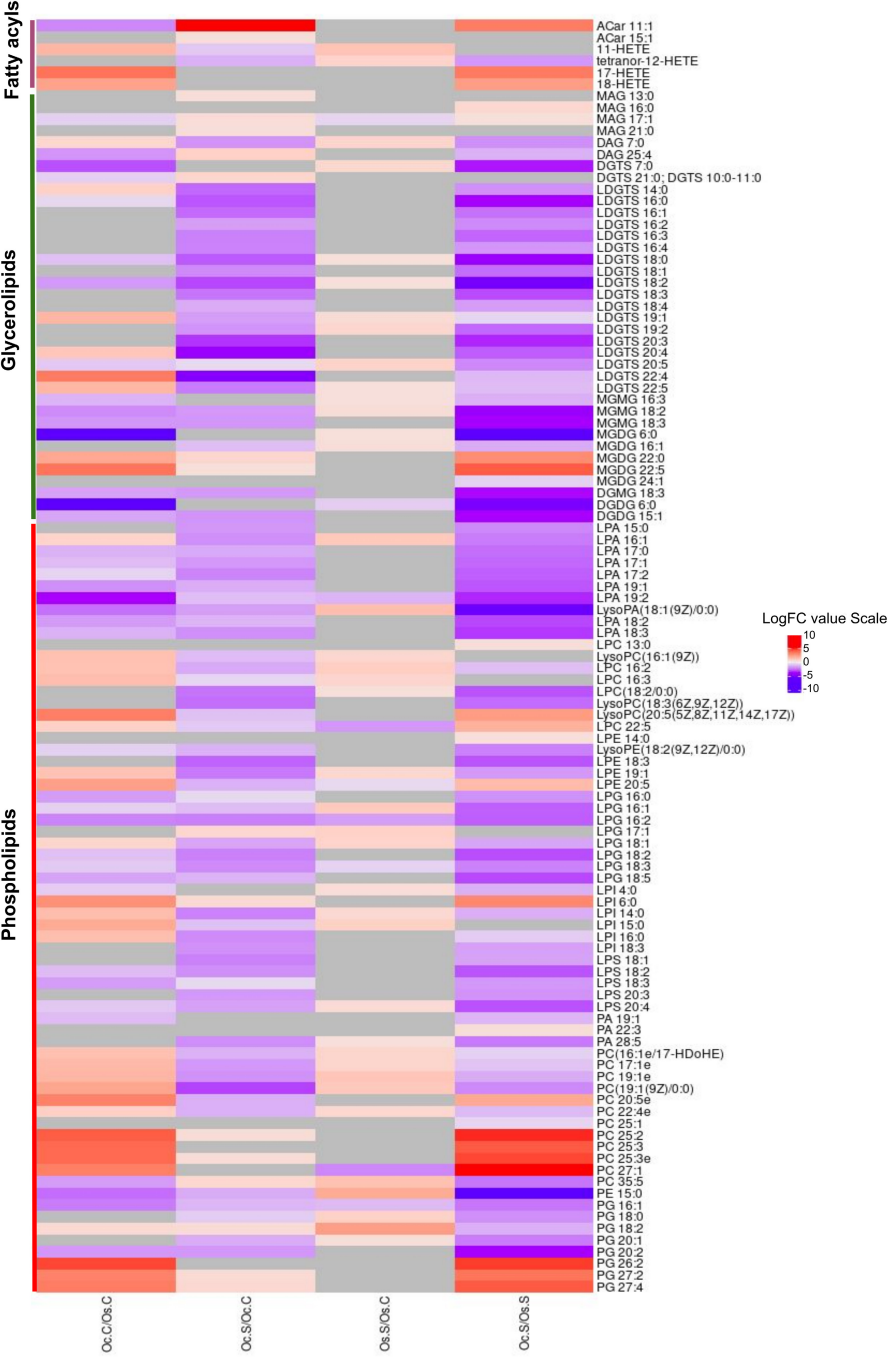
Heatmap analysis depicting the logarithm of fold change values for lipids in four comparison groups: Oc.C/Os.C, Oc.S/Oc.C, Os.S/Os.C and Oc.S/Os.S. Only lipids that showed differential expression (|Fold change|>1.5 and *p* <.05) in at least one of the four comparison groups were included. Heatmap cells with |Fold change|≤1.5 are shown in grey, indicating no significant change.

Analyzing the 2nd group of comparison (Oc.S vs Oc.C), we found no significant change in the concentration of xanthin compounds in *O. coarctata* when it underwent salt stress (File S3). Among the amino acids, 8 showed constitutive abundance in *O. coarctata* under control conditions. Among them, leucine, phenylalanine, and tyrosine were further upregulated by salt stress (Figure 5). In fact, leucine was exclusively upregulated in *O. coarctata* under stress (Figure 5). In the glycerolipid group, most of the lysodiacylglyceryltrimethylhomoeserine (LDGTS), monogalactosylmonoacylglycerol (MGMG), digalactosylmonoacylglycerol (DGMG), and digalactosyldiacylglycerol (DGDG) were found to be significantly downregulated in salt‐stressed *O. coarctata* samples compared to their control counterparts. In the phospholipid group, a similar trend was observed. Here, the majority of the lyso‐phosphatidylglycerol (LPG), lyso‐phosphatidate (LPA), and lyso‐phosphatidylserine (LPS) were also significantly downregulated under salt stress (Figure 6).

For the 3rd group of comparison (Os.S vs Os.C), the levels of astaxanthin and cassostreaxanthin B were observed to only be slightly upregulated (by 3.3⨯) in salt‐stressed *O. sativa* compared to their corresponding control samples (File S3). Apart from these xanthin compounds, three amino acids were upregulated in *O. sativa* under stress. They are phenylalanine, tyrosine, and tryptophan (Figure 5). In *O. sativa*, tryptophan was exclusively upregulated in salty conditions. However, *O. sativa* samples undergoing salt stress showed no significant change in most of the glycerolipids, although slight upregulation was observed in only a few of them in the Os genotype (Figure 6). In addition, salt stress did not affect the level of most of the phospholipids in *O. sativa*; although some nonsignificant up‐ or downregulation was observed (Figure 6).

For the final group (Oc.S vs. Os.S), we found that itaconate, PG (phosphatidylglycerol), and tyrosine were significantly upregulated in *O. coarctata* compared to *O. sativa*, under salt stress. This finding is consistent with the observed constitutive concentrations of the aforementioned metabolites in the genotypes' untreated control (Oc.C vs Os.C). This indicates that these metabolites might play an important role in conferring salt resistance in *O. coarctata*. Under salt stress, gelsemine, allantoin, benzyl alcohol, PE 15:0 (phosphatidylethanolamine), and prodiamine were found to be downregulated in *O. coarctata*, compared with *O. sativa* (Figure 4d). In the case of amino acids, a greater abundance of tyrosine was observed in *O. coarctata*, and phenylalanine was found to be induced by salt in both genotypes (Figure 5). In contrast to the first comparison group (Oc.C vs Os.C), phospholipid groups, such as lyso‐phophatidylinositol (LPI) and lyso‐phophatidylcholine (LPC), were downregulated under stress in *O. coarctata* but upregulated in *O. sativa* (Figure 6). Under salt stress, Lyso‐phophatidylethanolamines (LPEs) was downregulated only in *O. coarctata*, although these showed mixed profiles in the comparison group of Oc.C vs Os.C. Phosphatidylcholines (PCs) and phosphatidylglycerols (PGs) showed an interesting trend in their differential accumulation across the genotypes. Salt caused low‐carbon PCs to be downregulated while high‐carbon ones were upregulated in *O. coarctata* when compared to the situation in *O. sativa*. The same trend was observed under stress for low‐ and high‐carbon PGs in *O. coarctata* (Figure 6).

### 2.4 Pathway enrichment and topological analysis for differentially accumulated metabolites

Pathway enrichment analysis was done with the Pathway Analysis module of MetaboAnalyst 5.0 (https://www.metaboanalyst.ca/), and the KEGG Pathway database was used for further exploration. File S4 contains all the perturbed pathways for the four comparison groups.

**FIGURE 7:**
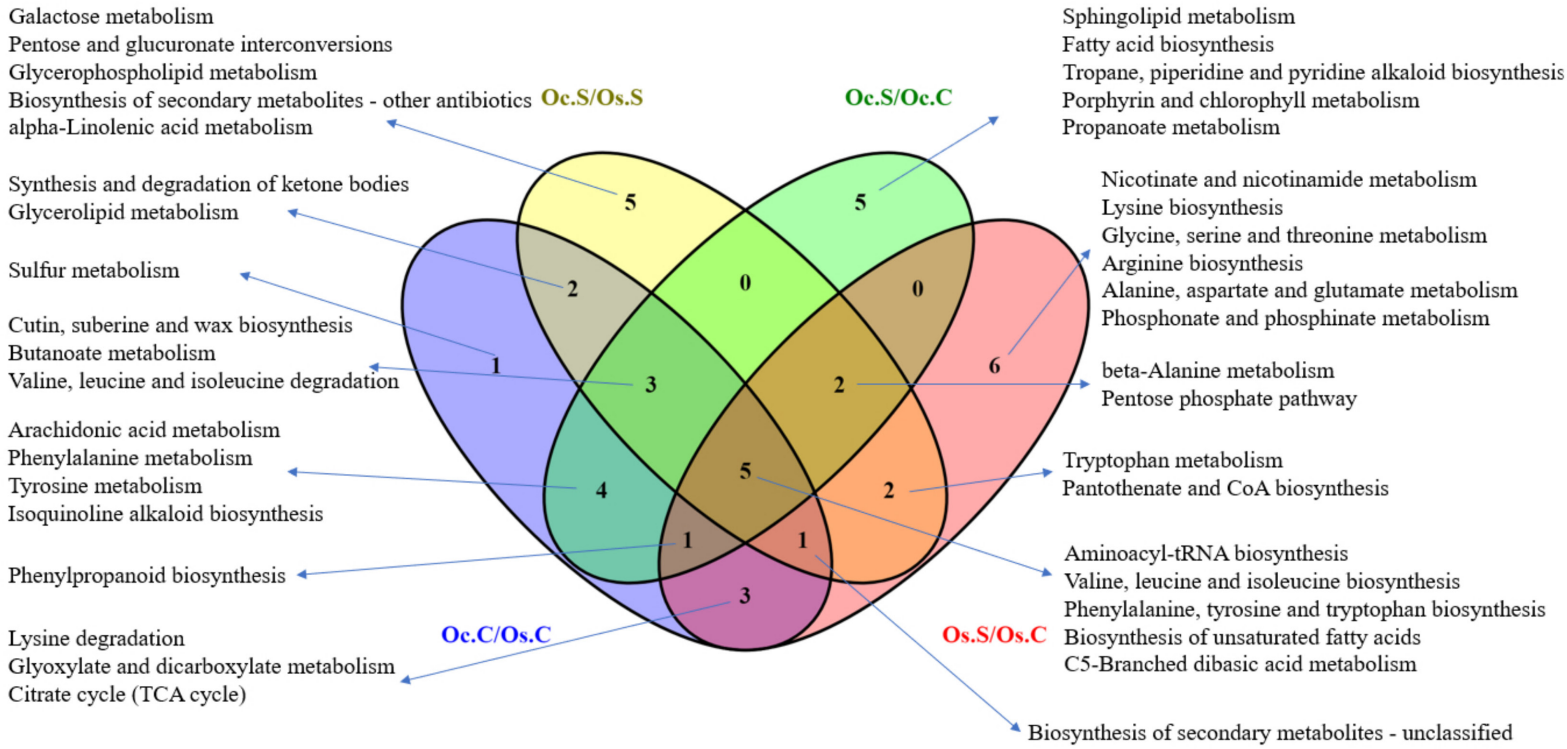
Venn diagram of top 20 KEGG pathways in which differentially accumulated metabolites (|fold change|>1.5 and *p* < .05) were involved for the 4 comparison groups: Oc.C/Os.C, Oc.S/Oc.C, Os.S/Os.C and Oc.S/Os.S.

**FIGURE 8:**
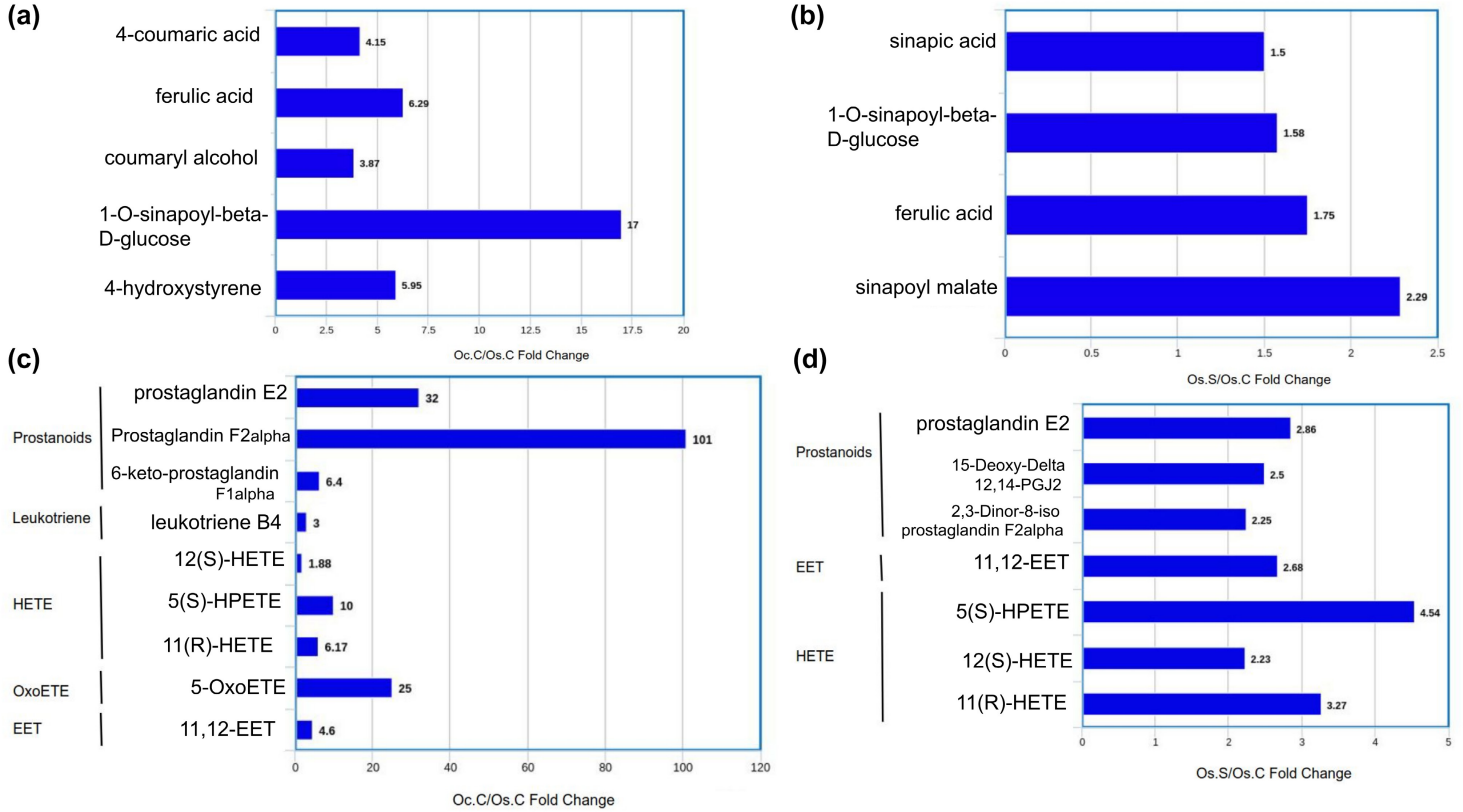
Bar graph representing (a) Oc.C/Os.C fold change of phenylpropanoids and their derivatives, (b) Os.S/Os.C fold change of phenylpropanoids, (c) Oc.C/Os.C fold change of eicosanoids, and (d) Os.S/Os.C fold change of eicosanoids.

We found that 5 metabolic pathways were significantly activated in all of the comparison groups (Figure 7). All of these were related to the metabolism of different types of amino acids, fatty acids, and carbohydrates. Secondary metabolite biosynthesis pathways were altered in both genotypes under salt‐stressed conditions. Sphinganine, sphingosine, and phytosphingosine metabolites seem responsible for the upregulation of sphingolipid metabolism in salt‐stressed *O. coarctata*. The phenylpropanoid biosynthesis pathway as well as cutin, suberin, and wax biosynthesis were more enriched in *O. coarctata* than in *O. sativa* under control conditions. Several phenylpropanoids and their derivatives were found in higher concentrations in *O. coarctata* (Figure 8a; File S4). When *O. sativa* is not exposed to salt stress, it does not express significant levels of phenylpropanoids. However, in the presence of salt stress, there is an increased accumulation of specific phenylpropanoids (Figure 8b).

Most of the eicosanoids detected in this study, including prostanoids, leukotrienes, HETE, OxoETE, and EET, were found to have accumulated in higher concentrations in *O. coarctata* compared to *O. sativa* (File S3). This indicates an upregulation of arachidonic acid metabolism in *O. coarctata* samples compared to that in *O. sativa*. In the face of salinity stress, *O. sativa* triggers a lower level of upregulation in the production of most of these eicosanoids, including several types of prostanoids, EET and HETE (Figure 8d; File S3). However, prostaglandin E2 and 12(S)‐HETE were downregulated in *O. coarctata* under similar conditions (File S3).

Biosynthesis of unsaturated fatty acids, nicotinate, and nicotinamide metabolism, lysine biosynthesis and degradation, and beta‐alanine metabolism were significantly influenced by the appearance of salt in *O. sativa* samples. For these pathways, *O. coarctata* showed slight or no alteration when exposed to salt stress. In *O. sativa*, the unsaturated fatty acids‐ (9Z)‐Octadecenoic acid, eicosapentaenoic acid, and linoleate were upregulated, while octadecanoic acid was downregulated (File S3). Most of the metabolites in the nicotinate and nicotinamide metabolism pathway and lysine degradation pathway were oarctata upregulated in *O. sativa* under stress In the beta‐alanine metabolism pathway, L‐aspartate, 3‐ureidopropionate, and pantothenic acid were shown to also be upregulated. Beta‐alanine metabolism helps to increase the level of pantothenic acid, which is a component of coenzyme A (CoA). TCA cycle along with glyoxylate and dicarboxylate metabolism were also upregulated in the salt‐stressed *O. sativa* compared to their control as 2‐oxoglutarate, cis‐aconitate, and citrate got upregulated. In the early salt stress response of *O. sativa*, the phenylpropanoid biosynthesis pathway was impacted by alterations in four metabolic intermediates, including phenylalanine, ferulate, sinapic acid, and p‐coumaric acid. The activity of the pentose phosphate pathway was shown to decrease only in *O. coarctata* when exposed to salt stress, as evidenced by a decrease in the levels of two of its intermediate products, D‐ribose and sedoheptulose 7‐phosphate (File S3).

## 3 DISCUSSION

The wild rice species, *O. coarctata* has survived environmental perturbations and adjusted its genetic profile over thousands of years to survive and set grains in seawater (40 dS/m). Therefore, its gene pool can be used to improve the tolerance and yield of domesticated rice at higher than the current levels of salinity in which it can grow (Tasnim et al., 2023; Tong et al., 2023). The high salt tolerance of this variety is possibly linked to many naturally adapted phenotypic changes, such as unicellular trichomes (Flowers et al., 1990), salt glands in both upper and lower leaf surfaces, and rhizoid‐like thin rootlets (Maisha et al., 2022; Sengupta & Majumder, 2010). At the gene level, the MIPS coding gene (*Pc INO1*) of *Oryza coarctata* is unique among all the *INO1* homologs known today (Majee et al., 2004), and functional introgression of the PcINO1 has conferred salt tolerance to a wide range of organisms, including crop plants (Das‐Chatterjee et al., 2006). In addition, *IF1* and *V‐ATPase* have also been suggested to be involved in the salt tolerance mechanism of *O. coarctata* (Mahalakshmi et al., 2006; Senthilkumar et al., 2005). Under saline conditions, *O. coarctata* can maintain a low leaf Na^+^/K^+^ ratio by accumulating more Na^+^ in the root rather than shoot (Prusty et al., 2018), suggesting that the root plays an important role in conferring salinity resistance in this species. Metabolic adaptation is also expected to function in coping with the high NaCl load. Metabolic profiling is currently employed as a means to investigate the underlying mechanism of defense against the salty environmental stress. This metabolic change is expected to reflect the ability of a specific genotype to adapt to high salinity stress in real time (Patel et al., 2022). The complete metabolic profiling of *O. coarctata* under salt stress has not been performed before. Moreover, no tissue‐specific comparative profiling data are available, although roots of *O. coarctata* likely play a major role in defending against salt stress as well as conferring tolerance through accumulating more sodium. In the present study, we, therefore, performed comparative metabolomic profiling of the roots of *O. coarctata* and *O. sativa* to facilitate our understanding of the changes taking place at the cellular level in the roots as they encounter salt stress, which subsequently allows it to adjust to an altered physiological status.

### 3.1 *O. coarctata* shows a distinct metabolite profile under control conditions

Several amino acids such as cysteine, valine, lysine, leucine and tyrosine were found in higher concentration in *O. coarctata* than in *O. sativa*. Moreover, salt‐stressed *O. coarctata* samples showed downregulation of methylmalonate and acetoacetate indicating slower degradation of valine, leucine, and isoleucine. High amino acid retention in the halophyte *O. coarctata* is possibly linked to its osmotic adjustment. This idea is substantiated by the fact that arabidopsis plants subjected to osmotic stress are shown to accumulate more leucine, isoleucine, and valine (Huang & Jander, 2017).

Exogenous vanillic acid (VA) was shown to improve salinity tolerance and plant growth performance by inducing the plant antioxidant defense in salt‐stressed tomato seedlings. Moreover, VA exhibited a protective effect against the accumulation of the toxic compound methylglyoxal (MG) by inducing the glyoxalase detoxification system under salt stress (Parvin et al., 2020). The enhanced glyoxalase system facilitates the conversion of excess MG to D‐lactate (Talaat & Todorova, 2022). Interestingly, our study revealed a significantly higher amount of vanillic acid in *O. coarctata* (>670‐fold) compared to *O. sativa*. This suggests that vanillic acid could be an important regulator of the salt stress response pathway in *O. coarctata*.

A good number of xanthin compounds, which are mainly carotenoids and work as ROS quenchers (Vladimirov, 1998) were found to be present in higher concentrations in *O. coarctata*. Klyachko‐Gurvich et al. (2000) explained how some carotenoids protect lipids and fatty acids of photosynthetic membranes from being oxidized by intense light stress‐generated ROS and help chloroplasts to maintain their membrane fluidity, permeability, and activity of their chlorophyll–protein complexes and polypeptide enzymes (Klyachko‐Gurvich et al., 2000). Ren et al. (2021) summarized optimal salt conditions that evoke different microalgal species to accumulate carotenoids and increase their survivability by protecting cells as antioxidants (Ren et al., 2021). *Oryza coarctata* was observed to maintain xanthin levels at a higher concentration even without salt stress and this phenomenon might help it to constitutively fight salt stress as a halophyte.

Increased allantoin, whether accumulated in vivo or externally applied, has been shown to reduce the endogenous H_2_O_2_ and O^2−^ concentrations under salt stress (Irani & Todd, 2016). Allantoin accumulation was increased in response to salt stress and ABA (abscisic acid) treatment in roots of tolerant and susceptible varieties of rice (PL177 and IR64, respectively). This accumulation was shown to be higher in tolerant genotypes (Wang et al., 2016). We report a contrasting finding that salt‐sensitive *O. sativa* has higher levels of allantoin (34.87×) compared to *O. coarctata*. Allantoin, a stress‐related purine metabolite, can activate jasmonate (JA) signaling in a MYC2‐regulated and abscisic acid‐dependent manner (Takagi et al., 2016). High concentrations of jasmonate can lead to over‐activation of ROS release and eventually cause cell death, which is likely what happens in glycophytes. Tolerant species are known to have quicker and more efficient depolarization‐activated NSCCs (nonselective cationic channels) than hyperpolarization‐activated NSCCs, leading to efficient management of ROS. On the other hand, hyperpolarization‐activated NSCCs can cause a delay in the generation and dissipation of salinity‐triggered stimuli. The delay can cause a sustained upregulation of ROS which leads to the unconstrained activation of Jasmonate signaling culminating in cell death (Ismail et al., 2014).

Under stress conditions, threonic acid is oxidized to threonate (Parsons et al., 2011) and an increase in the concentration of the latter helps to maintain cellular osmolarity. Muscolo et al., (2015) showed that the lowest level of threonic acid was present in the most stressed lentil genotype because most of it had been converted to the osmolyte, threonate (Muscolo et al., 2015). We observed that *O. coarctata* had a 10 times greater reservoir of threonic acid than *O. sativa* and under salt stress it was downregulated to seven times. Presumably, the threonic acid was being converted to its derivative osmolyte, threonate. Therefore, it is likely that *O. coarctata* is using its pool of threonic acid as a precursor for protecting itself against salt stress and the comparative lack of this precursor in *O. sativa* precludes its use of this strategy.

The differential metabolite profile between *O. sativa* and *O. coarctata* is also reflected in their KEGG pathway analyses. Glyoxylate, dicarboxylate metabolism, and TCA cycle were seen to be more activated in the *O. coarctata* than in *O. sativa*. Key components of these cycles that were found to be upregulated were 2‐oxoglutarate, citrate, and acetate. Several studies (Galili et al., 2016; Nunes‐Nesi et al., 2013) indicate that carbon skeletons from TCA cycle intermediates provide support to amino acid biosynthesis. Furthermore, amino acids are also known to be catabolized into precursors or intermediates of the TCA cycle for generating cellular energy for plant growth (Hildebrandt et al., 2015). Glyoxylate and dicarboxylate metabolism directly helps to regulate the balance of TCA cycle intermediates. These observations are consistent with the higher amino acid accumulation in *O. coarctata*.

By deamination, hydroxylation, and frequent methylation, phenylalanine generates coumaric acid and other acids with phenylpropane (C6‐C3) units, which are known as phenylpropanoids. Reduction of these acids results in some monolignols (alcohols) that are the starting compounds for the biosynthesis of lignin. We found several phenylpropanoids and their derivatives to be in higher concentration in *O. coarctata*. This aligns with the previous finding that *O. coarctata* contains more lignin in its composition compared to *O. sativa*. Maisha et al. (2022) showed that *O. coarctata* roots are protected by double‐layered lignified hexagonal epidermal cells of 67 μm thickness, whereas *O. sativa* roots contain non‐lignified double‐layered elongated cells with a thickness of around 53 μm (Maisha et al., 2022). It was shown that lignin deposition helps plants maintain cell turgor and fight drought stress by making cells more thickened and less permeable to water (Dong & Lin, 2021). Taken together, the observed differences in phenylpropanoids and their derivatives in *O. coarctata* suggest that increased root lignification may help the latter combat salt stress.

Phenylpropanoids that are derived from tyrosine and phenylalanine lead to the production of feruloyl‐CoA, which, along with unsaturated fatty acids, increase cutin, suberin, and wax biosynthesis in *O. coarctata*. This was supported by our observation of higher levels of ferulate (by 19×), oleic acid (3.6×), and 16‐hydroxy hexadecanoic acid (1.81×) in *O. coarctata*, accounting for the anatomical differences that set it apart as a halophyte. These observations are consistent with the leaf's phenotype of *O. coarctata* which are waxy unlike those of cultivated rice (Maisha et al., 2022).

### 3.2 Salt stress induces differential metabolite response in O. sativa and *O. coarctata*

More metabolite changes were observed in *O. sativa* compared with *O. coarctata* under salt stress. Nicotinate and Nicotinamide metabolisms were upregulated in salt‐stressed *O. sativa*. This pathway is responsible for regulating the level of NAD^+^ and NADP^+^ coenzymes which are redox‐active components and crucial for maintaining many metabolic pathways. They can confer biotic and abiotic stress tolerance by playing a rate‐limiting role in balancing reactive oxygen species. Consistent with this role, exogenous application of nicotinic acid was shown to enhance drought tolerance in Arabidopsis by boosting the salvage pathway of NAD biosynthesis (Ahmad et al., 2021). It is apparent that with all the other factors, especially constitutive mechanisms helping *O. coarctata* to alleviate stress, as discussed above, this mechanism may not be significant in the latter.

Lipids are a major constituent of plasma membranes, and some of them also serve as sensors and signaling molecules in important metabolic events (Liang et al., 2023). Previous studies have found that plant species contrasting in salt tolerance usually have different plasma membrane lipid profiles, and under saline conditions, membrane lipids of salt‐tolerant species are modulated in a way that favors the sustainability of membrane structure and functions to cope with the stress (Mansour et al., 2015). In this current study of metabolites at the whole root tissue level, we have shown many fatty acyls, glycerolipids, and phospholipids to maintain remarkably different profiles between *O. coarctata* and *O. sativa*. The increase in fatty acid levels in *O. coarctata* was particularly evident in comparison with *O. sativa*. An increase in fatty acid synthesis has been reported for the halophytic ice plant *Mesembryanthemum crystallinum* L. This facilitates the alteration of lipid biosynthesis to maintain membrane homeostasis and their physiological function under salt stress (Guo et al., 2022). The decrease in phospholipids under stress we observed is probably the result of sampling after 72 h. It has been reported that phospholipids signal the initiation of membrane lipid reconstruction (Han & Yang, 2021). On the other hand, organic acids have been reported to play a potential role in pH homeostasis in plants (Chen et al., 2009). It is not clear why the sensitive *O. sativa* shows induction of organic acids under stress since the pH of the soil in our experimental setup was acidic, around 6. The enhancement of arachidonic acid metabolic pathways that was observed only in *O. coarctata* has been reported in the halophytic grass, *Puccinellia nuttalliana* (Vaziriyeganeh et al., 2021). Interestingly, exogenous application of arachidonic acid was shown to enhance panicle health and grain filling in rice (Zainuddin et al., 2019). Our reported decrease in the concentration of glycerolipids is supported by the profile observed in the halophyte *Sueda salsa* (Sui et al., 2010). These researchers opine that the decrease in the content of MGDG and DGDG in *Sueda salsa* may cause a change in the bilayer‐forming lipid (DGDG) to an inverse hexagonal‐forming lipid (MGDG) (DGDG/MGDG ratio), which in turn affects the structure and micro‐viscosity of membranes. It is thus notable that all of the above‐mentioned metabolites were found to remodel in an entirely variable manner in the halophyte versus the glycophyte following salt exposure.

Phenylpropanoids and their derivatives were found to accumulate more in salt‐stressed *O. sativa*. Interestingly, these metabolites showed higher comparative abundance in untreated *O. coarctata*. As mentioned earlier, lignification of the root by the use of these phenylpropanoid precursors could provide a defense mechanism for the roots of wild rice against salt stress. This idea is consistent with the finding that salt and osmotic stress induce lignin accumulation through activating NAC transcription factors and E2 ubiquitin‐conjugating enzyme 34 (PtoUBC34) (Dong & Lin, 2021). The increased abundance of these metabolites in *O.coarctata* under normal conditions provides a clue as to how *O. coarctata* is always primed to fight salt stress. In addition, biosynthesis of cutin, suberin, and wax fueled by existing levels of phenylpropanoid compounds was found to be further elevated in *O. coarctata* to combat salt stress. Whether this distinct lipid profile plays a crucial role in the higher salinity tolerance trait of *O. coarctata* or not requires further research.

## 4 CONCLUSION

The present study of root tissue‐specific comparative metabolite profiling revealed a preexisting defense mechanism against salt stress in the halophyte *O. coarctata*. These include maintaining an osmotic balance by greater levels of branched‐chain amino acids and threonic acid. In addition, there are mechanisms in place to maintain the level of xanthin and enhanced levels of monolignols and phenylpropanoids to maintain lignin, cutin, suberin, and wax biosynthesis. Last but not least, *O. coarctata* lowers the levels of methylglyoxal, a toxic byproduct that is greatly enhanced under salt stress. Therefore, using a combination of phenotypic appendages and a multiple‐tiered metabolic defense, *O. coarctata* can thrive in seawater. The exemplified metabolic shifts provide a valuable basis for comparative evaluation of other salt‐tolerant rice and other varieties. The most promising adaptations could then be employed to enhance salt defense strategies in cultivated rice varieties.

Although previous studies have detected the differential accumulation of many of these metabolites in separate instances and different species, *O. coarctata* possesses them collectively as a complete machinery to withstand salt stress as a halophyte. Only a few studies have delved deeper into the intricacies involved in the altered level of metabolites and the corresponding enhancement in stress tolerance demonstrated by this plant. Thus, a more extended study of how the identified metabolites can contribute to increased stress tolerance may help identify components of metabolic pathways that could be potentially manipulated. With advancements in technologies for CRISPR‐Cas mediated alteration in multiple target genes, it may be possible to metabolically engineer heightened salt tolerance and resilience in commercial rice genotypes, surpassing current achievements in dealing with the negative impact of climate change.

## 5 METHODS

### 5.1 Plants growth condition and treatment

The experiment was conducted in the natural environment in a netted enclosure of the Plant Biotechnology Laboratory, University of Dhaka. The day and night temperatures were, respectively, 34 ± 3 and 27 ± 2°C and the relative humidity 75 ± 5%. The test species were salt‐sensitive *O. sativa* (cultivar BRRI dhan28) and salt‐tolerant *O. coarctata*. *O. sativa* seeds were washed with tap water, then rinsed once before being soaked in distilled water for 72 h and placed in a 37°C incubator. The seeds were then transferred to styrofoam sheets floating in trays containing Yoshida's solution (Gregoria, 1997). *O. coarctata* does not easily grow from seeds, and therefore, seedlings with rhizomes were separated from plants previously growing in pots and immersed in Yoshida's solution in the same type of tray as the *O. sativa*. The hydroponic solution was changed every 2 days. When *O. sativa* grew to the three‐leaf‐and‐one‐bud stage, 60 mM NaCl stress was applied to the stress trays of both species. Then, salt concentration was increased by 20 mM each day until the treatment group reached 120 mM. The purpose of this gradual increase in salinity was to avoid salt shock to the plants. *O. coarctata* grows in 400 mM salt, whereas rice seedlings are sensitive above 40 mM salt. However, the recommended concentration of salt for screening rice seedlings by the International Rice Research Institute is 100–120 mM salt (Gregoria, 1997). In order to have a comparative picture between *O. sativa* and *O. coarctata* we therefore chose 120 mM. Control trays were adjusted with an equal volume of hydroponic solution without salt. Isolation of the metabolites was done in triplicate. There were four groups of root tissue in total: *O. sativa* control (Os.C), *O. sativa* salt‐stressed (Os.S), *O. coarctata* control (Oc.C) and *O. coarctata salt‐stressed* (Oc.S), each of which consisted of three separate samples.

### 5.2 Metabolite extraction

In total, 500 mg of each root sample was weighed and crushed by mortar pestle according to the protocol for extraction provided by the Company, Creative Proteomics Inc. to which samples were sent for metabolite analysis (De Vos et al., 2007). Samples were then transferred to tubes with 600 μL of 80% methanol, and vortexed for 90 s followed by sonication for 30 mins at 4°C. Then, samples were kept at −20°C for 1 h, vortexed for 30 s, and kept at 4°C for 15 min, followed by centrifugation at 10,000 rpm at 4°C for 20 min. 200–500 μL of supernatant was collected and stored at −80°C overnight. Samples were then dried in a freezer the next day. Processed samples were shipped to the commercial service provider, Creative Proteomics, Inc (Shirley, NY, USA) for analysis of the total untargeted metabolites. For the metabolomic analyses, three biological replicates for each genotype under both control and salt‐stressed conditions were used.

### 5.3 LC–MS

Quality control (QC) samples were used to evaluate the methodology. The separation was performed by ACQUITY UPLC (Waters) combined with Q Exactive MS (Thermo) and screened with ESI–MS. The LC system consisted of ACQUITY UPLC HSS T3 (100 × 2.1 mm × 1.8 μm) with ACQUITY UPLC (Waters). The mobile phase was composed of solvent A (0.05% formic acid water) and solvent B (acetonitrile) with a gradient elution (0–1 min, 5% B; 1–12 min, 5%–95% B; 12–13.5 min, 95% B; 13.5–13.6 min, 95%–5% B; 13.6–16 min, 5% B). The flow rate of the mobile phase was 0.3 mL·min^−1^. The column temperature was maintained at 40°C, and the sample manager temperature was set at 4°C.

Mass spectrometry parameters in ESI+ and ESI− mode are listed as follows:

*ESI+*: Heater Temp 300°C; Sheath Gas Flow rate, 45 arb; Aux Gas Flow Rate, 15 arb; Sweep Gas Flow Rate, 1arb; spray voltage, 3.0KV; Capillary Temp, 350°C; S‐Lens RF Level, 30%.

*ESI−*: Heater Temp 300°C, Sheath Gas Flow rate, 45 arb; Aux Gas Flow Rate, 15 arb; Sweep Gas Flow Rate, 1arb; spray voltage, 3.2KV; Capillary Temp,350°C; S‐Lens RF Level,60%.

### 5.4 Data analysis

The raw data was acquired and aligned using the Compound Discover 3.1 software (Thermo Fisher Scientific, Waltham, MA, USA). Standard/default parameters for analyses were used as advised by the Analytical Company, Creative Proteomics, Inc. After normalizing to total peak intensity, metabolites from both ESI‐ and ESI+ were merged and imported into the Metaboanalyst 5.0 (Pang et al., 2021) for downstream analysis, including principal component analysis, hierarchical clustering analysis, and making volcano plots of differentially accumulated metabolites combining results of fold change analysis and *T*‐tests. The KEGG pathway database (https://www.genome.jp/kegg/pathway.html) was additionally used to explore the metabolomic pathways. Venn diagrams were created using the Venny 2.1 interactive tool (https://bioinfogp.cnb.csic.es/tools/venny/). The heatmap of the lipids were formed using in‐house R script. One‐way ANOVA followed by Šídák multiple comparisons test was performed for comparing amino acid profiles using GraphPad Prism version 9.4.1 for Windows, GraphPad Software, Boston, Massachusetts USA, www.graphpad.com.

## FUNDING INFORMATION

This work was supported by the Bangladesh Climate Change Trust (BCCT).

## CONFLICT OF INTEREST STATEMENT

The authors declare no conflict of interest.

## Supporting information


**File S1:** Metabolites in *Oryza sativa* and *Oryza coarctata* root.

**File S2:** Venn diagram results for differentially expressed metabolites.

**File S3:** Differentially accumulated metabolites.

**File S4:** List of enriched pathways by differentially accumulated metabolites.

**Figure S1:** The morphology of *Oryza coarctata*.

## Data Availability

The data underlying this article are available in the article and its online supplementary material.
