# Supplementary figures and images for: Comparative metabolite profiling of salt sensitive Oryza sativa and the halophytic wild rice Oryza coarctata under salt stress
Source: Plant Environ Interact. 2024 Jun 15;5(3):e10155. doi: 10.1002/pei3.10155 (PMC11179383; doi:10.1002/pei3.10155)

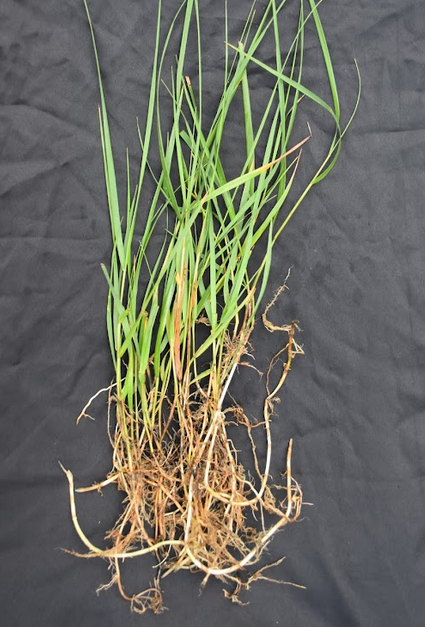

Supplement: Supplementary file 5 — Figure S1: The morphology of Oryza coarctata. [file PEI3-5-e10155-s003.png]
